# Structural Analysis of an Epitope Candidate of Triosephosphate Isomerase in *Opisthorchis viverrini*

**DOI:** 10.1038/s41598-018-33479-8

**Published:** 2018-10-10

**Authors:** Jonghyeon Son, Sulhee Kim, So Eun Kim, Haemin Lee, Myoung-Ro Lee, Kwang Yeon Hwang

**Affiliations:** 1 Division of Biotechnology, College of Life Sciences & Biotechnology, Korea University, Seoul, 136-701 South Korea; 2 Division of Malaria & Parasitic Disease, Korea National Institute of Health, Osong, 28159 Republic of Korea

## Abstract

*Opisthorchis viverrini*, a parasitic trematode, was recategorized as a group 1 biological carcinogen because it causes opisthorchiasis, which may result in cholangiocarcinoma. A new strategy for controlling opisthorchiasis is needed because of issues such as drug resistance and reinfection. Triosephosphate isomerase (TIM), a key enzyme in energy metabolism, is regarded as a potential drug target and vaccine candidate against various pathogens. Here, we determined the crystal structures of wild-type and 3 variants of TIMs from *O. viverrini* (OvTIM) at high resolution. The unique tripeptide of parasite trematodes, the SAD motif, was located on the surface of OvTIM and contributed to forming a 3_10_-helix of the following loop in a sequence-independent manner. Through thermal stability and structural analyses of OvTIM variants, we found that the SAD motif induced local structural alterations of the surface and was involved in the overall stability of OvTIM in a complementary manner with another parasite-specific residue, N115. Comparison of the surface characteristics between OvTIM and *Homo sapiens* TIM (HsTIM) and structure-based epitope prediction suggested that the SAD motif functions as an epitope.

## Introduction

*Opisthorchis viverrini*, a prevalent human liver fluke, is a parasitic trematode that is widely distributed in Southeast Asia^1^. After infection, *O. viverrini* resides in the bile ducts and causes minor symptoms such as inflammation and loss of appetite. Thus, the risk of opisthorchiasis has been underestimated and the disease has been regarded as minor. Many *in vivo* studies have shown that opisthorchiasis is a cause of cholangiocarcinoma (CCA)^2–4^. Additionally, recent studies showed that the excretory-secretory products (ESP) of *Clonorchis sinensis*, which is a related fluke that is also involved in cholangiocarcianoma, are related to oncogenesis^5^. In fact, the International Agency of Cancer Research reclassified *O. viverrini* as a group 1 biological carcinogen in 2009^6^.

Since praziquantel (PZQ) was developed in the 1970s, chemotherapy involving PZQ has been a major strategy for controlling zoonotic helminthiasis such as opisthorchiasis, schistosomiasis, clonorchiasis, and echinococcosis. Although the overall prevalence and infection levels of zoonotic helminthiasis was slowly reduced, new issues have arisen. Because of its powerful efficacy and safety, PZQ was used exclusively for approximately three decades, which resulted in the appearance of resistance and reinfection. For example, reinfection by *O. viverrini* after PZQ treatment was reported in northeast Thailand in 2016^7^. Furthermore, it was reported that repeated administration of PZQ elevated the risk of CCA^8^. For *Schistosoma mansoni*, a parasitic flatworm that causes schistosomiasis, the development of drug resistance has been suggested several times at the laboratory and worldwide levels^9–12^. Therefore, a new strategy for controlling and preventing helminthic diseases should be established, such as developing additional anthelminthic drugs and vaccines.

Triosephosphate isomerase (TIM, EC 5.3.1.1) is a key enzyme in glycolysis and catalyzes the reversible isomerization between dihydroxyacetone phosphate (DHAP) and glyceraldehyde 3-phosphate (GAP). It was reported that chronic hemolytic anemia and severe neuromuscular disease was found in the TIM deficiency patients^13^ and DHAP was markedly accumulated in the erythrocytes of the patients^14^. Given that glycolytic enzymes are essential for maintaining life, some reports have suggested that the indispensability of glycolytic enzymes can be used in developing a drug or vaccine to overcome not only clonorchiasis^15–17^ but also other zoonotic helminthiasis^18–20^. It was reported that a unique tripeptide existed in TIMs from parasitic flatworms but not in those from other species, including non-pathogenic free-living planarians and vertebrates^20,21^. Although the reason why SXD/E tripeptide (X represents Ala, Ile, or Lys) exists in only parasitic flatworms is unclear, this motif clearly differs between the pathogen and its host. Therefore, molecular structural identification of the pathogen-specific SXD/E motif in OvTIM may be useful for vaccine development. Here, we determined the crystal structure of wild-type and 3 variants of OvTIMs at high resolution and confirmed the different formation of the following rigid secondary structure compared to that of human TIM. Additionally, we identified the stabilizing function of residue N115 and performed structural analysis of the surface characteristics of OvTIM to assess whether the SAD motif functions as an epitope.

## Results

### Overall structure of OvTIM

**Figure 1:**
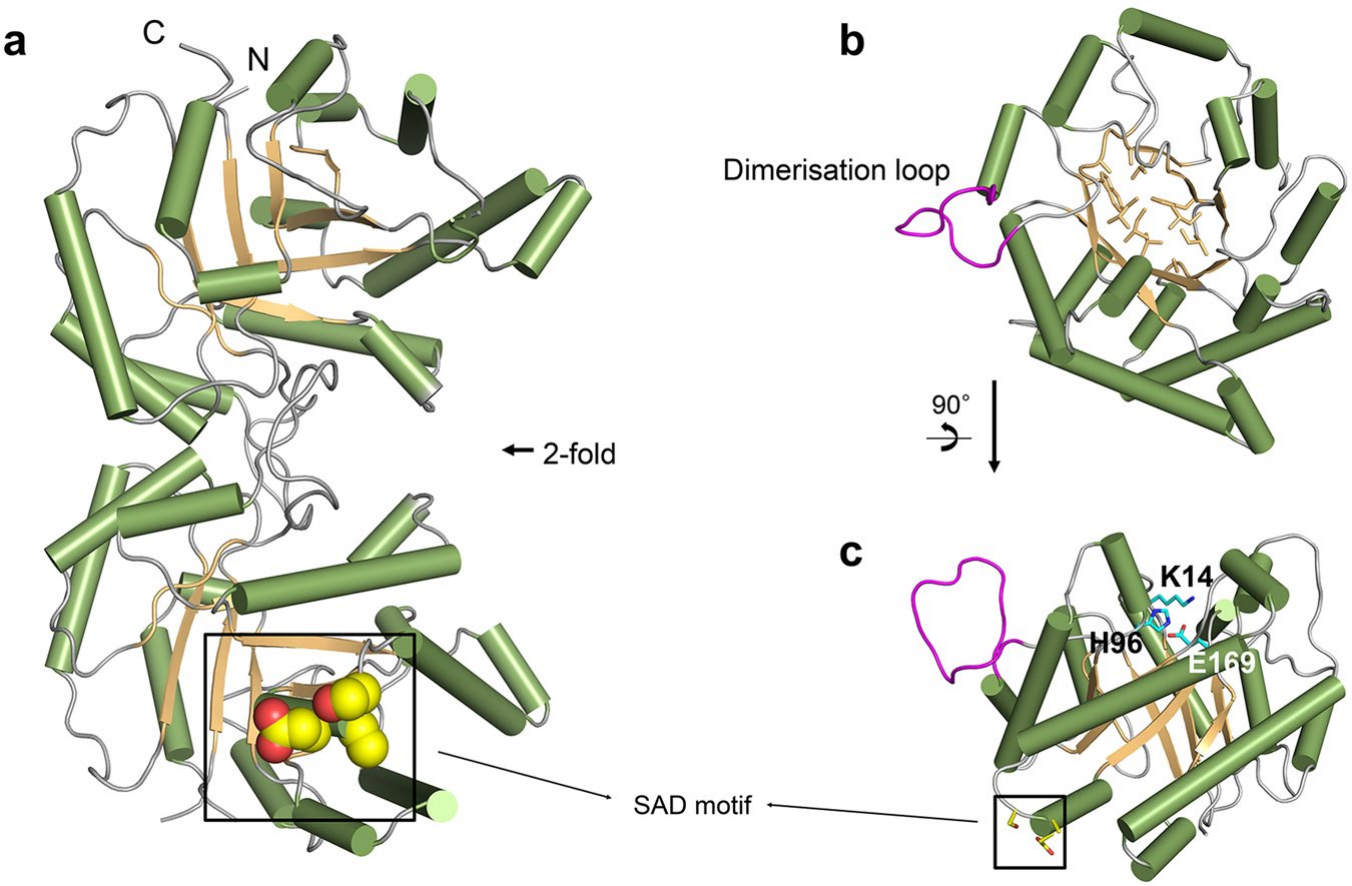
Overall structure of OvTIM. (**a**) Overall dimeric structure of OvTIM. The bundle of α-helices and β-barrel of OvTIM are represented as *green* and *orange* cartoon models, respectively. The SAD motif is denoted as a *yellow* sphere model. (**b**) The top view and (**c**) rotation view of the monomer of OvTIM are displayed. Hydrophobic residues forming the β-barrel fold are represented as a stick model. The catalytic triad, SAD motif, and dimerization loop are highlighted as *cyan, yellow*, *and magenta*, respectively.

**Figure 2:**
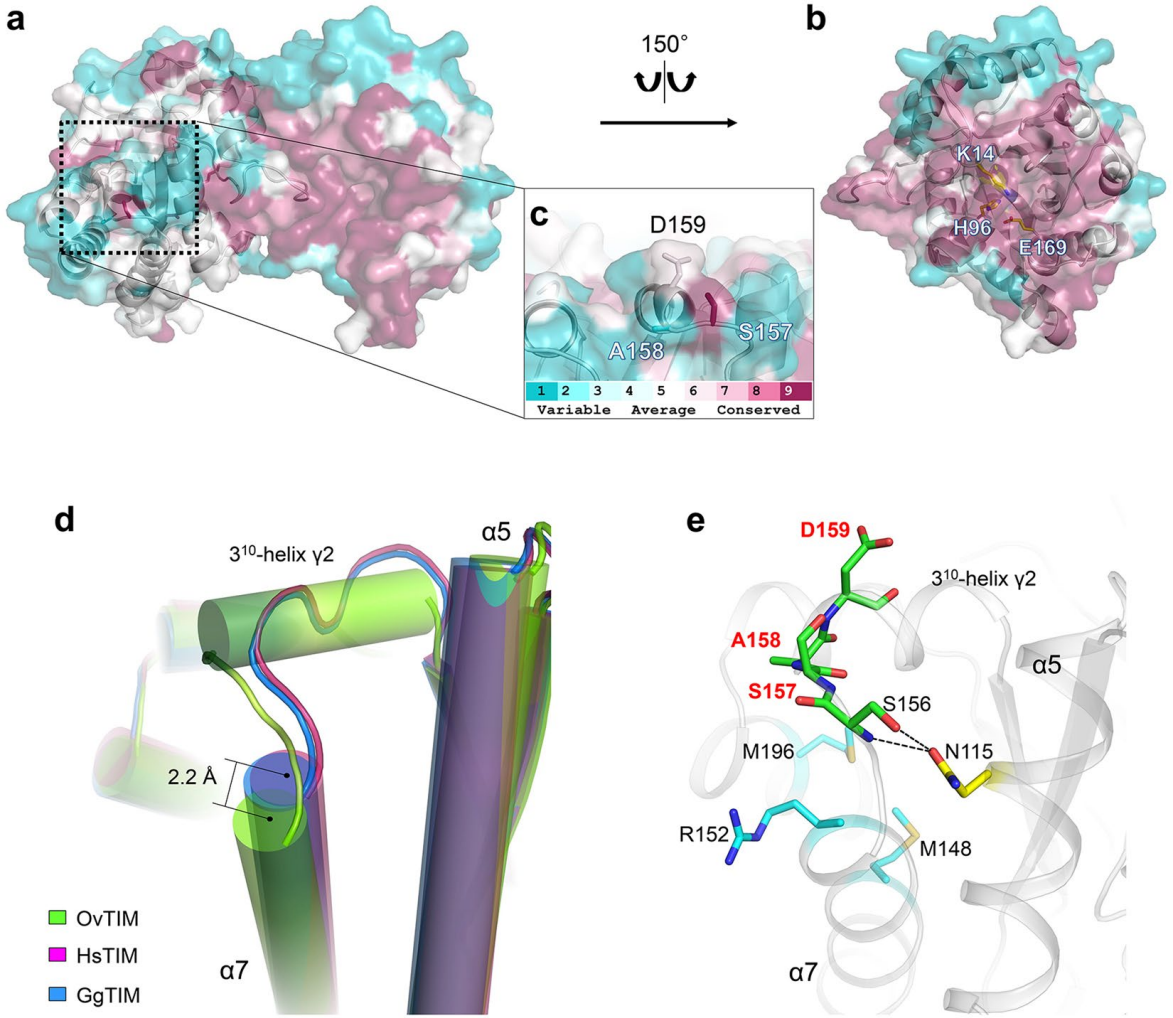
Structural analysis of SAD motif. (**a**) Degree of residue conservation of TIM among 7 parasitic trematodes (*O. viverrini* (accession code: A0A074Z863), *Clonorchis sinensis* (accession code: G7YDG0)*, Schistosoma haematobium* (A5A6F9)*, Schistosoma japonicum* (Q27775)*, Schistosoma mansoni* (P48501)*, Schistosoma turkestanicum* (Q45XG1), and *Taenia solium* (Q9GTX8)) is represented as color variations in a surface model from *cyan* (lowest conserved residue) to *wine* (highest conserved residue). (**b**) Rotated view of the OvTIM monomer is shown, and residues forming the catalytic triad are represented as *yellow* sticks. (**c**) An enlarged view of the SAD motif. (**d**) Superimposition of OvTIM (*green*) structure with *Homo sapiens* TIM (PDB code: 4POC) and *Gallus gallus* TIM (GgTIM) (PDB code: 8TIM) is represented in *magenta* and *blue*, respectively. The corresponding region of the SAD motif in OvTIM is designated as *dark green*. (**e**) The intermolecular interaction of the region around the SAD motif. The bulky side chains are highlighted as a *cyan* stick model.

We determined the crystal structure of OvTIM and other variants at high resolution by molecular replacement. The amino acid residues except for residues 175–179 in OvTIM showed clear electron density. Two homodimers in the asymmetric unit were identical with an r.m.s. deviation Cα of 0.22 Å. The overall structure of OvTIM is similar to that of other known TIMs in that it contains 12 α-helices, three 3_10_-helices, and eight parallel β-sheets, forming a classical α/β barrel fold (Fig. 1 and Supplementary Fig. S1). The hydrophobic core of the inner β-barrel is stabilized by hydrophobic residues such as F8, L41, A43, A63, W91, I93, I125, V165, I211, and L234. The inner β-barrel is protected from solvents by a bundle of α-helices (Fig. 1b,c). The solvent-accessible area of each subunit and dimer interface area are 19,728 Å^2^ and 1,609.8 Å^2^, respectively. The loop between α2 and β3 interacts with 2 loops of another protomer (β1 and α1, α3 and β4) to participate in dimerization of OvTIM. The residues involved in the dimerization and catalytic activity are highly conserved among species (Fig. 2a,b). The loops in which catalytic residues are located form dimerization interface with the dimerization loop of another protomer, indicating that OvTIM shares a common mechanism with triose phosphate isomerization based on the dimer formation. The catalytic triad (K14, H96, and E169) is in the upper entrance of the β-barrel, while the SAD motif is on the surface opposite the catalytic site and exposed to the outside of the protein, as expected.

### Structural analysis of the SAD motif

According the multiple sequence alignment results, Ser, the first residue in the SXD/E motif, is almost conserved among 7 parasitic trematodes except for *Clonorchis sinensis* TIM (CsTIM) (Fig. 2c and Supplementary Fig. S1). The second position of the SXD/E motif is mainly occupied by a hydrophobic residue except for *Taenia solium* TIM (TsTIM) and CsTIM, while an acidic amino acid (Asp, Glu) is in the third position^21^. In OvTIM, a specific SAD motif (S157–D159) is located between α7 and β6, inducing the formation of a subsequent 3_10_-helix γ2 (D159–I164), although the corresponding region is not conserved in either vertebrate TIMs (Fig. 2d and Supplementary Fig. S2). Two *in silico* structures of TIMs from *S. mansoni*^20^ and *Fasciola hepatica*^22^ was reported. The overall fold was well predicted to OvTIM, r.m.s. deviation of 0.44 Å (SmTIM) and 0.47 Å (FhTIM) for Cα, respectively. However, the back-bone assignment and parasite-specific 3_10_-helix induced by SXD/E motifs of *in silico* structures were not matched to the SAD motif in OvTIM (Supplementary Fig. S3). The side chains of the SAD motif that protrude into the solvent region are not well-defined, while surrounding hydrophobic residues such as L155, M160, and W161 are directed towards the core of the protein to take part in the hydrophobic interaction (Fig. 2e). Additionally, the N115 in α5 is another characteristic feature of OvTIM. In parasitic trematode TIMs, polar residues such as Asn, Glu, Gln, and Lys are found rather than Ala as in vertebrate TIMs (Supplementary Fig. S1). The side chain of N115 forms hydrogen bonds with the main chain and side chain of S156, stabilizing the loop located between α7 and γ2. The residues forming the hydrophobic core surrounded by α7, γ2, and α9 are also substituted with bulky amino acids such as M148, R152, and M196 in OvTIM. This substitution in OvTIM resulted in tilting of the end part of α7 toward the solvent region by ~2.2 Å because of steric hindrance of the substituted bulky side chain and N115 compared to that of the corresponding region of *Homo sapiens* TIM (HsTIM) (Fig. 2d,e). Therefore, formation of short 3_10_-helix γ2 caused by the SAD motif, the stabilizing function of N115, and tilting of α7 cause unique local alterations in the surface structure in OvTIM, distinguishing it from vertebrate TIMs.

### Local structural alteration and overall stability affected by the SAD motif and N115

**Figure 3:**
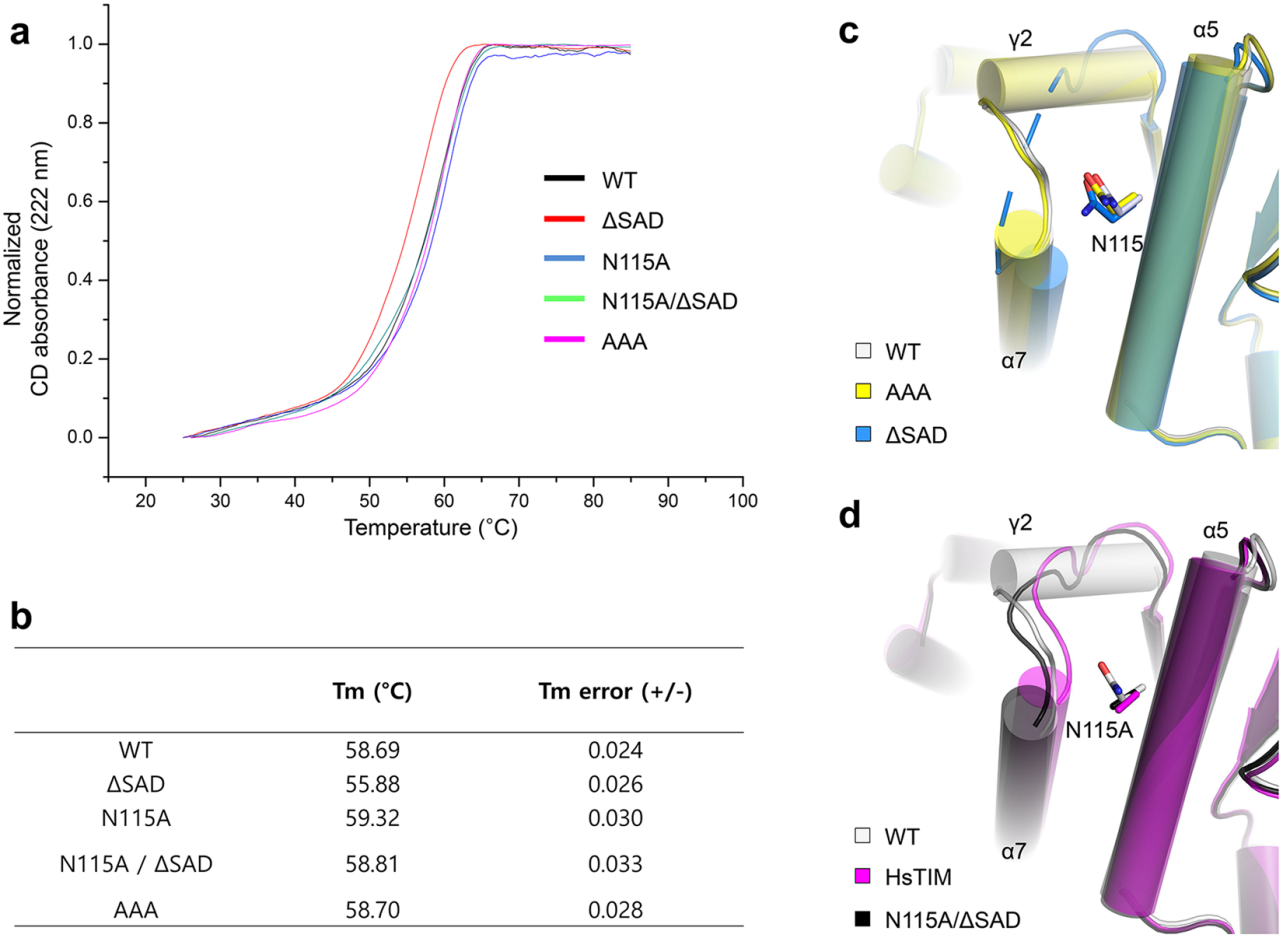
Thermostability of OvTIM and variants and comparative analysis. (**a**) Thermal stability of wild-type and variants (AAA, ∆SAD, N115A, and N115A_∆SAD). (**b**) Melting temperature of variants based on the CD absorbance at 222 nm. (**c**) Superimposition of wild-type, AAA, and ∆SAD variant structures. N115 residues from wild-type, AAA, and ∆SAD are represented as a stick model. The disordered region of ∆SAD variant is represented as a dash. (**b**) Superimposition of the OvTIM, HsTIM, and ∆SAD_N115A structures.

To estimate the effect of the parasite unique tripeptide on the overall protein stability of OvTIM, we performed circular dichroism to measure the thermal stability of various OvTIM variants (Fig. 3a,b). We constructed four variants, including a SAD deletion variant (ΔSAD), variant in which the SAD region was substituted with AAA (AAA), variant in which N115 was replaced with Ala (N115A), and double variant (N115A_ΔSAD). First, the thermal shift of the melting curve of the AAA variant was negligible, indicating that the side chains of the SAD motif that protrude into the solvent region do not contribute to protein stability. Interestingly, the ΔSAD variant exhibited a lower protein thermal stability, with a Tm of ~3 °C, compared to that of the wild-type. In contrast, the Tm of the N115A variant was slightly elevated, with a Tm of approximately 0.63 °C above the wild-type Tm, while the Tm of N115A_ΔSAD was restored to that of the wild-type. Structural analysis of variants was also carried out to evaluate the effects of the unique structural alteration in OvTIM (Fig. 3c,d). In the ΔSAD variant, the corresponding region (K153–W158) was not defined except for the molecule of the region which is stabilized via an interaction with other symmetry molecules, while the AAA variant showed a similar structure as the wild-type. The electron density of N115 of ΔSAD was not defined and normalized B factor of N115 in ΔSAD structure and wild-type structure were 42.71 Å^2^ and 16.01 Å^2^, respectively. These results show that the hydrogen bond between N115 and the main chain of S156 no longer forms because of deletion of the SAD motif. Consequently, the remaining steric hindrance of N115 pushes the α7 so that α7 exhibits a more shifted orientation than that of the wild-type. The α7 of N115A_ΔSAD variant moves to the original position of the wild-type and exhibits a stable conformation because all other steric hindrance caused by N115 does not exist. The overall shape of the designated region of the N115A_ΔSAD variant is similar to that of HsTIM but showed a unique difference with an α7 shift that resulted from the bulky side chains (M148, R152, and M196). Therefore, the SAD motif and intramolecular interaction of N115 as well as the bulky side chains are major stabilizing factors in OvTIM.

### Comparison of surface characteristics between OvTIM and HsTIM

**Figure 4:**
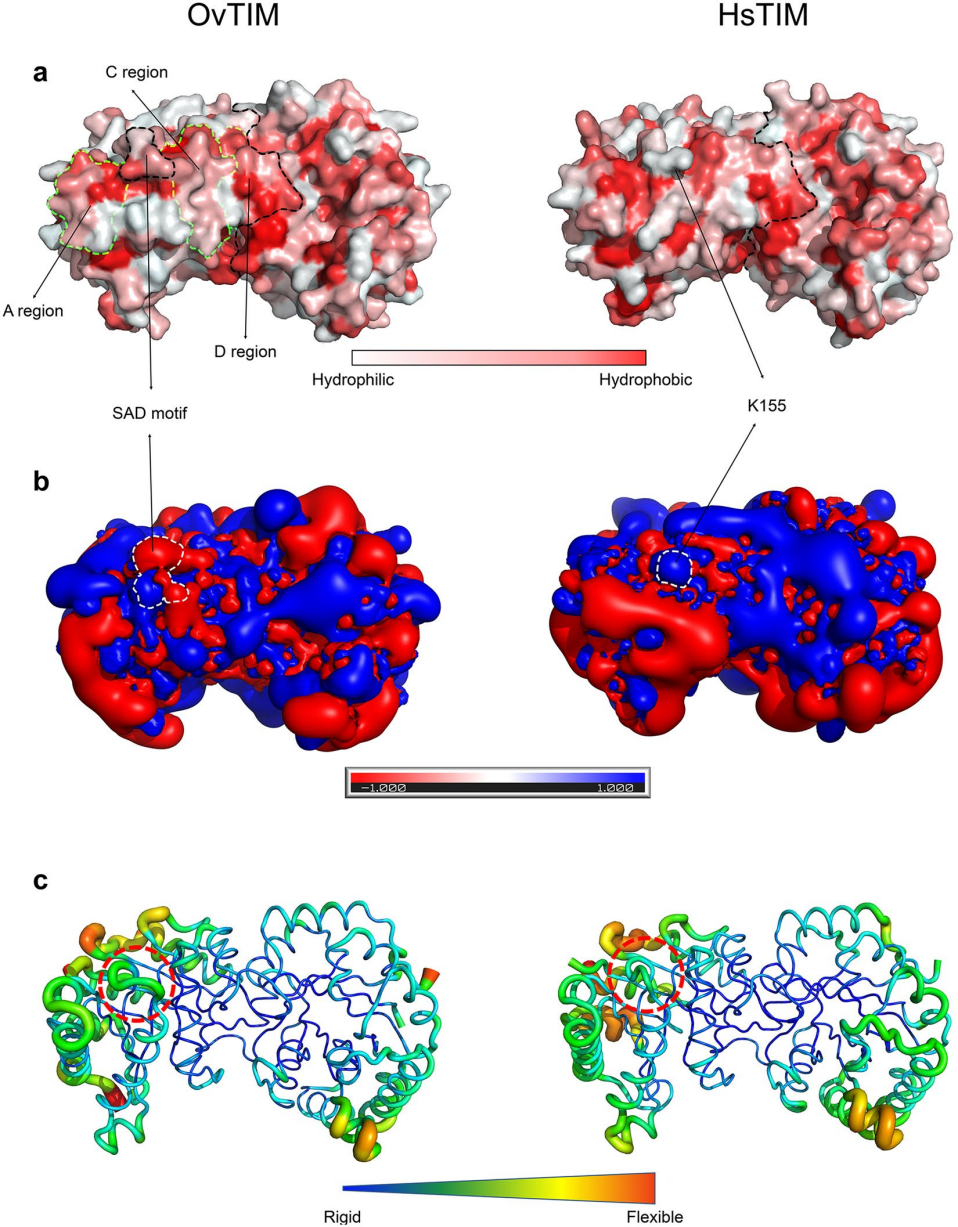
Comparison of surface characteristics between OvTIM and HsTIM. (**a**) Hydrophobicity of the 4 hydrophobic regions comprising the long hydrophobic patch in OvTIM is represented as dotted lines. (**b**) Electrostatic field, the SAD motif in OvTIM and K155 in HsTIM are designated as *white* dotted lines. (**c**) Temperature factor, the SAD motif in OvTIM and K155 in HsTIM are designated as *red* dotted circles.

The positions of Cα in OvTIM and HsTIM are identical, with an r.m.s.d. = 1.08 Å, showing great similarity. However, the surface characteristics are quite different between the two enzymes (Fig. 4). The total solvent-accessible surface area of OvTIM (19,728 Å^2^) is larger than that of HsTIM (18,931 Å^2^), while the hydrophobic surface area of OvTIM (5,355 Å^2^) is smaller than that of HsTIM (5,630 Å^2^). Interestingly, the cluster of hydrophobic residues in OvTIM results in formation of a long hydrophobic patch. The patch can be divided to 4 parts (Fig. 4a): a region which in the helix-turn-helix motif between α9 and α10, SAD motif region, B region located on the end part of α5, and C region on the exterior surface of the dimerization loop. In contrast, the K155 residue of HsTIM that is present rather than the SAD motif interrupts the consecutive hydrophobic patch. The distribution of the electrostatic fields of OvTIM is also distinct from that of HsTIM (Fig. 4b). Both electrostatic fields of HsTIM tend to be clustered exclusively, while OvTIM shows a relatively even electrostatic field distribution. Additionally, the SAD motif region shows another characteristic feature related to its charge distribution compared to the K155 region of HsTIM. The K155 region, which shows a moderate positive electrostatic field, is located between two strong electrostatic fields. The SAD motif, which has a more protruding D159 than the K155 of HsTIM, exhibits strong negative electrostatic fields. The distribution degree of the temperature factor in both TIMs is universal in that the external surface residues show relatively high flexibility (Fig. 4c). The side chain atoms of the SAD motif (45.4 Å^2^) showed a slightly high temperature factor compared to the corresponding region in HsTIM (30.9 Å^2^).

### Structure-based epitope prediction of OvTIM

**Figure 5:**
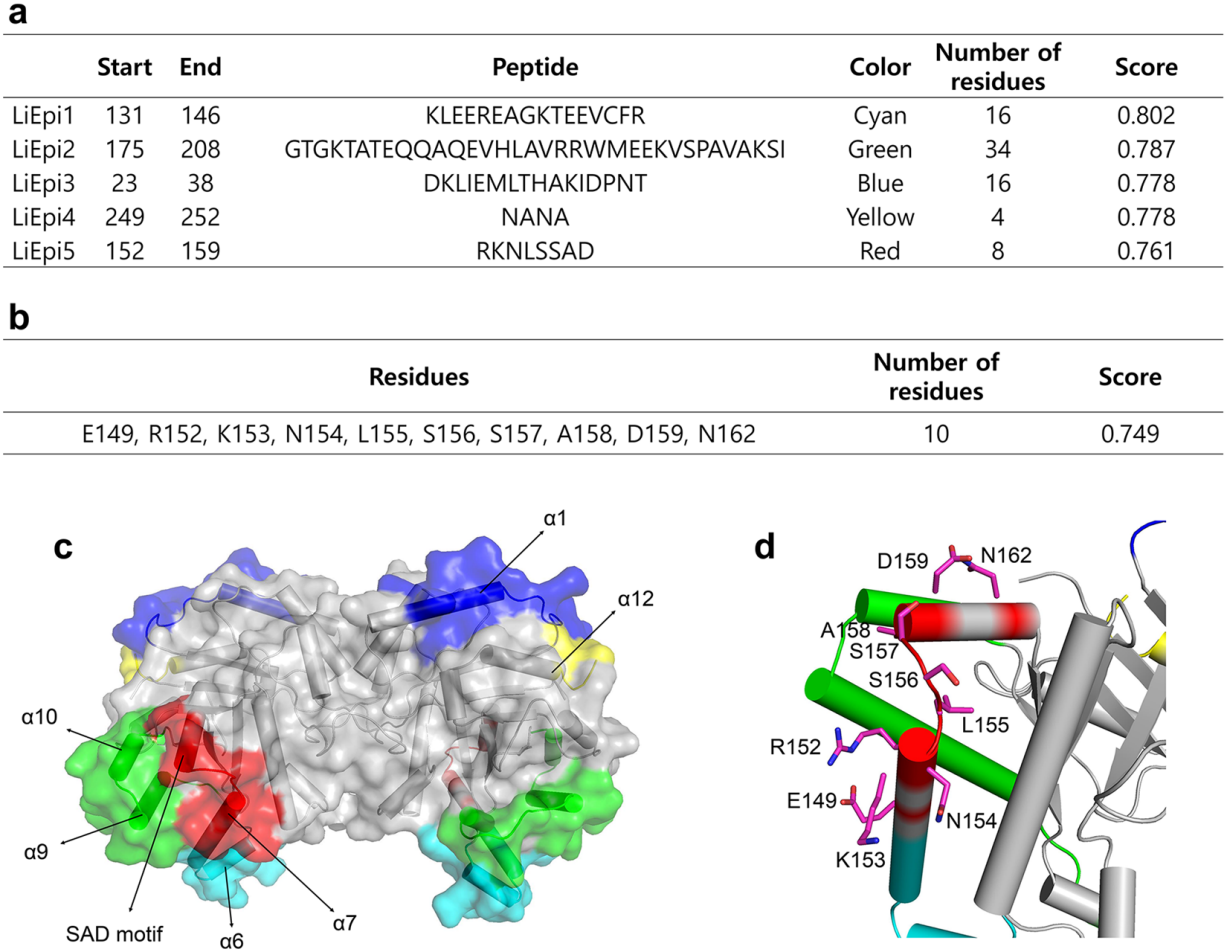
Structure-based epitope prediction of OvTIM. (**a**,**b**) Predicted linear epitope (**a**) and discontinuous epitope (**b**) from the ElliPro results. For precise results, the dimer structure of OvTIM was used, and possible epitopes with a score over 0.7 are listed. (**c**) The results of the predicted linear epitope in (**a**) are designated on the surface model of OvTIM. (**d**) The residues predicted to form discontinuous epitope are represented as a stick model.

We predicted the epitope on the OvTIM using ElliPro, which is a modified prediction program developed by Thornton to identify epitope regions that protrude from the globular surface of a protein^23^ (Supplementary Fig. S4). Among all possible continuous epitopes, a total of 5 potential epitopes with a score above 0.70 were predicted to be located at the surface region of OvTIM (Fig. 5a,c). The predicted linear epitopes are broadly classified into 2 regions; LiEpi3 which includes α1 and LiEpi4 which includes the end of α12 are located at the C-terminal part of OvTIM. The remaining linear epitopes clustered near the SAD motif, including α6 (LiEpi1), α9 and α10 (LiEpi2), and the SAD motif and α7 (LiEpi5). In contrast, only the SAD motif is included in the discontinuous epitope, was predicted by a modified method based on the protrusion index (S) and distance (R) calculation of the residue’s center of mass (Fig. 5b). In addition to the SAD motif, peripheral hydrophilic residues such as E149, R152, K153, N154, and N162 collaboratively participate in the protrusion (Fig. 5d).

## Discussion

In this study, we determined and analyzed the structure of OvTIM, which showed high similarity with other known TIMs, suggesting a common intrinsic mechanism. As previously reported^15–20^, inhibiting the parasitic glycolytic enzyme is advantageous. Since the similarity of the active site and reaction mechanism between the parasite and its host makes it challenging to develop drugs that selectively inhibit the parasitic enzyme, it is important to identify a new pathogen-specific inhibitory mechanism that is not shared with its host. It was reported that thiol-reactive reagents induced decreased affinity for the substrates by chemical modification of C222 of TIM from *Giardia lamblia* (GlTIM) that was located in the non-catalytic region^24,25^. Interestingly, the corresponding region of the C221 of OvTIM shows the similar environment with that of GlTIM, surrounding hydrophobic pocket is well conserved (Supplementary Fig. S5). Therefore, we may suggest that the inhibitory strategy based on thiol-reactive compound could be also possible in the case of OvTIM. The SAD motif, which is a parasite-specific region, is not included in the catalytic and conserved region. Furthermore, the efficiency of OvTIM ΔSAD to isomerase DHAP to GAP showed slightly lower than that of wild-type in the quick activity check assay (Supplementary Fig. S6). Considering them, the SAD motif may be another potential region of the parasite-specific inhibitory strategy.

We confirmed that the SAD motif induced formation of the 3_10_-helix γ2, which is a loop structure in vertebrate TIMs. The side chain of the SAD motif protruded into the solvent region and no significant Tm change was observed in the AAA variant, indicating that local structural alterations and the stability of OvTIM are related to the number of amino acids of the SAD motif and not the exact sequence of the SAD motif. We also found that the N115 residue, which is conserved as a bulky and hydrophilic residue in parasitic flatworms, is involved in forming the unique parasitic surface structure via hydrogen bonding with S156. The overall stability of OvTIM was also affected by the SAD motif and N115 in a complementary manner in that the Tm decrease in the ΔSAD variant was rescued by N115A substitution.

In contrast to the sequence-independent contribution of the SAD motif to the surface structure change and stability, the side chains of the SAD motif, which are exposed to the solvent region, are still open to participate in the host-parasite interaction. It has been reported that CsTIM and fructose-1,6-bisphosphate aldolase (EC 4.1.2.13) of *O. viverrini*, which were identified by immunoscreening with human CCA serum^26,27^, exist not only in the cytosol but also in the ESP^28^. Additionally, cell surface display of TIMs in various pathogens was observed^15,19,29–31^. In *Schistosoma mansoni*, a monoclonal antibody recognizing SmTIM was identified and specifically inhibited the reactivity of SmTIM^18^. These reports indicate the importance of glycolytic enzymes in zoonotic helminths and other pathogens in the host-parasite interaction as well as energy metabolism. From this perspective, we performed a surface characteristic comparison of OvTIM and HsTIM because the difference between a parasite and its host is a major factor affecting the host-parasite interaction. The distribution degrees of hydrophobicity, electrostatic field, and flexibility are major factors in the protein-protein interaction and antigen-antibody complex^32–34^. As a result, we identified a long hydrophobic patch in OvTIM, electrostatic field difference between OvTIM and HsTIM, and relatively high flexibility of the SAD motif. In fact, the hydrophobic patch, the center of which is the SAD motif, is surrounded by polar or charged residues, which is a major characteristic of the epitope. Moreover, we predicted using ElliPro^23^ that the SAD motif of OvTIM is a discontinuous epitope that is directly recognized by B-cell receptors. Together with the high flexibility and hydrophobicity of α9, the flexibility and high protruding index of the SAD motif suggest that the SAD motif forms an interface with the complementary-determining region of the antibody paratope. The linear epitope range from K148 to V161 in HsTIM, which includes the corresponding region of the SAD motif in OvTIM, was reported^35^ (epitope ID 438866) in Immune Epitope Database and analysis resource (IEDB). The difference in the charge distribution between the SAD motif in OvTIM and the reported epitope in HsTIM is included in another selection mechanism between the parasite and its host. Analysis of the surface characteristics and the fact that OvTIM is included in the ESP support that the SAD motif is an epitope. In conclusion, the results of this study provide a new pathway for vaccine development against *O. viverrini* and another strategy for overcoming drug resistance and reinfection.

## Methods

### Cloning and expression of OvTIM

cDNA encoding triose phosphate isomerase from *O. viverrini* was synthesized and amplified by PCR^5^. The amplified DNA was digested with the restriction nucleases NdeI and XhoI and cloned into the vector pET17b, which contained a 6 His-tag and thrombin site. The recombinant plasmid was transformed into *Escherichia coli* BL21 (DE3) pLysS cells for expression tests under various conditions. The soluble portion of the target protein was observed, and the target was scaled-up to increase expression. The clone was grown at 37 °C in LB medium until the OD600 reached 0.6–0.8. The target protein was induced by adding isopropyl β-D-1-thiogalactopyranoside at a final concentration of 1 mM at 18 °C for 18 h. The cells were harvested by centrifugation at 4 °C at 4,392 × *g* for 20 min, and the pellet was stored at −70 °C.

### Purification of OvTIM

The frozen pellet was resuspended in buffer A (20 mM Tris, pH 8.9, 50 mM NaCl, 2 mM β-mercaptoethanol) and disrupted by sonication on ice for 25 min. Cell debris was pelleted by centrifugation (24,878 × *g* for 60 min). The filtered supernatant was purified using a HisTrap HP column (GE Healthcare, Little Chalfont, UK). The recombinant protein bound on the column was eluted with a linear imidazole concentration gradient of buffer B (20 mM Tris, pH 8.5, 50 mM NaCl, 500 mM imidazole, 2 mM β-mercaptoethanol). The purity of the target protein was determined by SDS-PAGE, and fractions were pooled for further purification and 6x His-tag of recombinant protein was not cleaved. The protein solution was concentrated to 1 mL using Amicon Ultra centrifugal filters and then diluted with buffer C (20 mM Tris, pH 8.5, 2 mM DTT) up to 10 times to lower the salt concentration. The diluted protein solution was loaded onto a HiTrap Q HP column (GE Healthcare), and a linear NaCl gradient in buffer D (20 mM Tris, pH 8.5, 1 M NaCl, 2 mM dithiothreitol) was applied. Because two different surface ionic characteristics of OvTIM were observed in the SDS-PAGE results, each protein was concentrated separately. Size-exclusion chromatography was performed for each protein using a HiLoad 26/60 Superdex-200 prep-grade column (GE Healthcare) in final buffer (20 mM Tris, pH 7.5, 100 mM NaCl, 2 mM DTT). The purified proteins were finally concentrated to about 80 and ~40 mg/mL, respectively, and then stored at −70 °C.

### Crystallization

The initial crystallization conditions were identified by the sitting-drop vapor diffusion method using the commercial Index screening kit (Hampton Research, Aliso Viejo, CA, USA) in MRC 2 Well Crystallization Plates (Hampton Research). Next, 0.5 μL of solution that contained ~80 mg/ml of proteins was mixed with an equal volume of screening solution. Initial crystals were observed under these conditions (0.2 M magnesium chloride hexahydrate, 0.1 M Tris, pH 8.5, 25% polyethylene glycol 3350 at 293 K) 5 days later. A single crystal suitable for diffraction was obtained by mixing 1 μL of protein solution with an equal volume of reservoir solution (0.3 M magnesium chloride hexahydrate, 0.1 M Tris, pH 8.5, 22% polyethylene glycol 3350 at 20 °C) by the hanging-drop vapor diffusion method. The crystals were cryoprotected using 25% (v/v) glycerol before mounting, and then transferred to cryo-solution for a few seconds.

### Data collection and structure determination

The diffraction data set for the crystal of OvTIM were collected at beamline 5 C of the Pohang Light Source (PLS, Pohang, South Korea) at a wavelength of 1.00001 Å. Images were indexed, integrated, and scaled using HKL2000^36^. We carried out molecular replacement for phasing using the TIM of chicken (PDB entry 1TPH) as a model with the Phaser module in PHENIX^37^. Next, 95% of the residues were automatically built with the Autobuild module in PHENIX^38^, and building of the remaining residues was performed manually using Coot^39^. The model of OvTIM that was refined using the Refine module in PHENIX^37^ was validated using MolProbity^40^. The final values of R_work_ and R_free_ were 0.1618 and 0.1990, respectively. The other variants of OvTIM were determined by molecular replacement using the wild-type of OvTIM as a model. The statistics for data collection and refinement are provided in Table 1.

**Table 1:** Statistics for X-ray data collection and refinement.

	OvTIM_wild-type	OvTIM_ΔSAD	OvTIM_AAA	OvTIM_N115A_ΔSAD
Data collection				
X-ray source	PAL BL5C	Spring-8 BL44XU	PAL BL11C	PAL BL11C
Wavelength (Å)	1.00001	0.90000	0.97942	0.97942
Space group	C222_1_	P2_1_	P2_1_2_1_2_1_	P2_1_
Unit-cell parameters				
a, b, c (Å)	96.53 206.62 97.47	73.74 91.92 75.78	68.84 90.37 102.27	74.04 92.56 76.08
α, β, γ (°)	90 90 90	90 109.128 90	90 90 90	90 109.39 90
Resolution (Å)	50.00–1.75 (1.81–1.75)	50.00–1.75 (1.85–1.75)	50.00–1.58 (1.61–1.58)	50.00–1.80 (1.83–1.80)
R_merge_ (%)	10.5 (11.0)	7.4 (42.5)	16.9 (47.3)	9.1 (27.3)
<I/*σ*(I)>	64.05 (21.1)	14.81 (3.26)	15.15 (2.04)	26.38 (5.03)
Completeness (%)	98.8 (93.3)	99.7 (99.2)	98.7 (95.3)	97.2 (95.1)
Average multiplicity	5.6 (3.1)	4.24 (4.19)	7.9 (4.5)	5.3 (4.0)
Observed reflections	541,897 (28061)	410,026 (64976)	654,169 (17640)	464,383 (17084)
Unique reflections	96,825 (9052)	96,733 (15516)	82,985 (3920)	87,968 (4271)
Refinement				
R_work_/R_free_ (%)	0.1640/0.2004	0.1599/0.1904	0.1706/0.1950	0.1466/0.1810
Average B factor (Å^2^)	21.192	19.162	18.469	21.271
R.m.s. deviations				
Bond lengths (Å)	0.007	0.006	0.007	0.009
Bond angles (°)	0.929	0.975	0.937	1.104
Ramachandran plot (MolProbity^±^)				
Favored (%)	97.41	97.18	98.17	97.60
Allowed (%)	2.59	2.82	1.83	2.40
Outliers (%)	0	0	0	0
PDB code	5ZFX	5ZG4	5ZG5	5ZGA

### Site-directed mutagenesis

Four OvTIM variants, N115A, ∆SAD, AAA, and N115A-∆SAD, were produced using a QuickChange site-directed mutagenesis kit (Stratagene, La Jolla, CA, USA). The variants were expressed and purified as described above for wild-type OvTIM.

### Thermal shift assay

Each protein including the wild-type and variants were diluted to 0.5 mg/mL with buffer (20 mM HEPES, pH 7.5, 100 mM NaCl) prior to loading. The samples were loaded and then heated from 25 to 85 °C at 0.5 °C/min. The circular dichroism absorbance at 222 nm was recorded using a circular dichroism spectrophotometer (Jasco, Oklahoma City, OK, USA) and normalized to calculate the melting temperature of each protein.

### Structural analysis

The amino acid sequences of TIMs were aligned via CLUSTALW^41^, and the results were processed using the ESPript^42^ server. The surface model of the residue conservation degree was constructed using the Consurf^43^ server. The solvent-accessible surface area and buried area were calculated using PISA^44^ and the surface area that was composed of hydrophobic residues was added to calculate hydrophobic surface area. Protein topology diagrams which show protein secondary structures and fold were drawn using the Pro-origami^45^ server. The hydrophobicity of surface residues was calculated using the Eisenberg hydrophobic scale^46^. To precisely compare surface temperature (***B***) factors between OvTIM and HsTIM, the temperature factors were normalized using a previously described equation^47^:

$${B}_{{\rm{norrmalised}}}=[100-0]\times [B-{B}_{{\rm{\min }}}]/[{B}_{{\rm{\max }}}-{B}_{{\rm{\min }}}].$$

The electrostatic field and normalized temperature factor of proteins were calculated and visualized using PyMol^48^. All images describing the structures of proteins were made using PyMol^48^.

### Catalytic activity measurement

The catalytic activity of wild-type OvTIM and ΔSAD variant to isomerase DHAP to GAP was measured using triose phosphate isomerase activity colorimetric assay kit (BioVision)^49,50^. Absorbance at 450 nm was plotted to NADH concentration for standard curve after seven NADH solutions which range from 0.05 mM to 0.4 mM were incubated with enzyme mix and developer for 10 mins. The absorbance of reaction mixtures containing 0.4 mM DHAP and 0.018 mM OvTIM dimer was recorded at 450 nm in every 10 secs. Total 2 sets of activity curves of wild-type OvTIM and ΔSAD variant were obtained and plotted after conversion of the absorbance to NADH concentration based on previously determined standard curve.

## Electronic supplementary material

Supplementary Information
