# Supplementary material for: Structural Analysis of an Epitope Candidate of Triosephosphate Isomerase in Opisthorchis viverrini
Source: Sci Rep. 2018 Oct 10;8:15075. doi: 10.1038/s41598-018-33479-8 (PMC6180082; doi:10.1038/s41598-018-33479-8)
Supplement: Supplementary file 1 — Supplementary Information [file 41598_2018_33479_MOESM1_ESM.pdf]

Structural Analysis of an Epitope Candidate of Triosephosphate Isomerase in *Opisthorchis viverrini*

Jonghyeon Son[1,‡], Sulhee Kim[1,‡], So Eun Kim[1,‡], Haemin Lee[1], Myoung-Ro Lee[2], and Kwang Yeon Hwang[1,*]

[1]*Division of Biotechnology, College of Life Sciences & Biotechnology, Korea University, Seoul 136-701, South Korea*

[2]*Division of Malaria & Parasitic Disease, Korea National Institute of Health, Osong, Republic of Korea*

‡These authors contributed equally to this work.

*Corresponding author. Fax: +82 (0)2 923 3229. Telephone: +82 (0)2 3290 3512. E-mail address: chahong@korea.ac.kr (K.Y. Hwang)

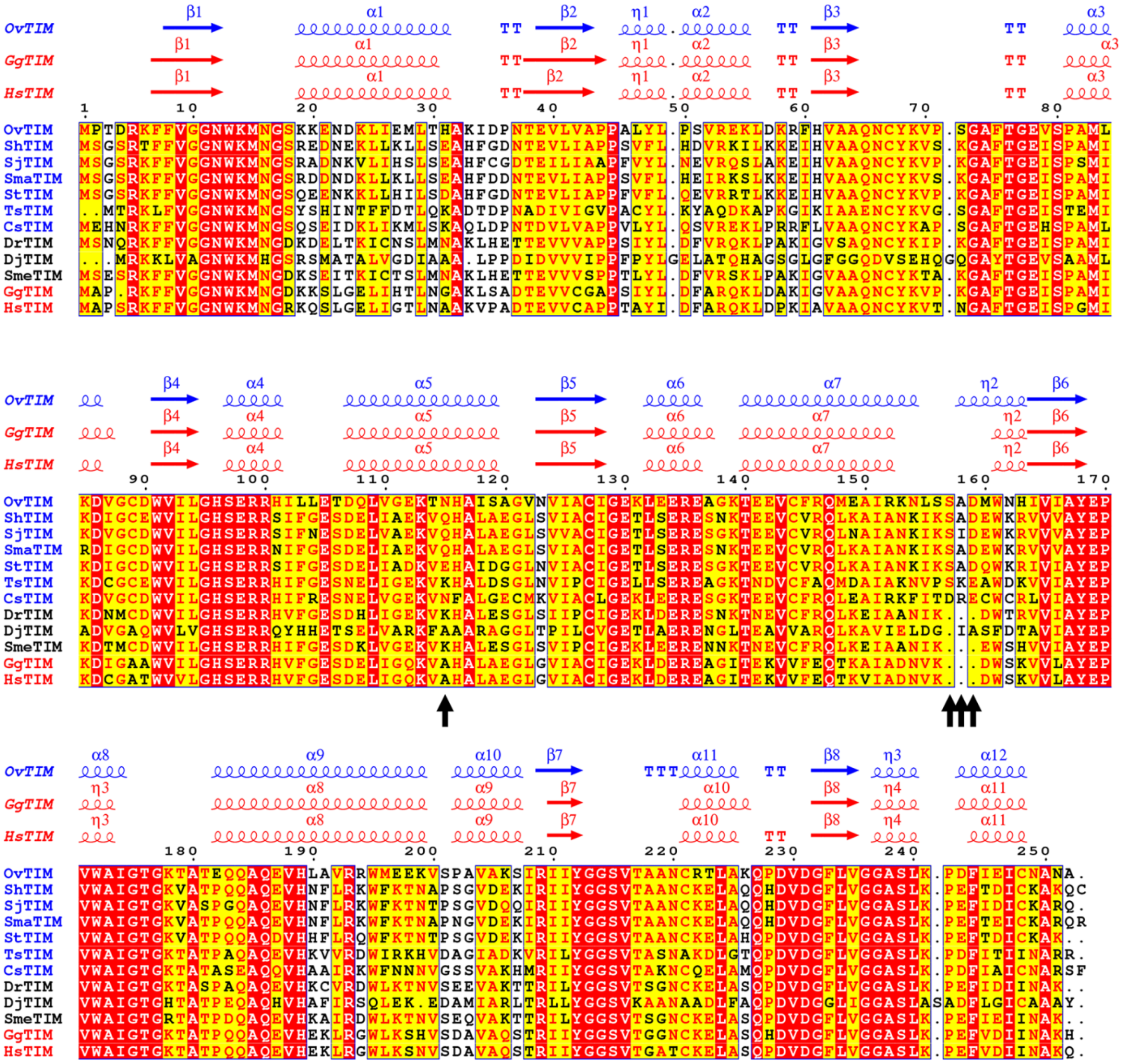

Supplementary Figure S1. Multiple sequence alignment of Triosephosphate isomerases (TIMs) from various species. TIMs from 3 free living planarian species (labelled in *black*) and 2 vertebrates (labelled in *red*) (*Dugesia ryukyuensis* (GenBank accession number BW639877), *Dugesia japonica* (GenBank accession number EU288186), *Schmidtea mediterranea* (GenBank accession number DN315650), *Gallus gallus* (P00940), and *Homo sapiens* (P60174-1)) are included in addition to 7 TIMs from parasite trematodes (*Opisthorchis viverrini* (accession code: A0A074Z863)*,* *Clonorchis sinensis* (accession code: G7YDG0), *Schistosoma haematobium* (accession code: A5A6F9), *Schistosoma japonicum* (Q27775), *Schistosoma mansoni* (P48501), *Schistosoma turkestanicum* (Q45XG1), and *Taenia solium* (Q9GTX8)). The secondary structures of OvTIM, GgTIM, and HsTIM are shown in the upper side of sequence. N115 and the SAD motif are highlighted as *black* arrows.

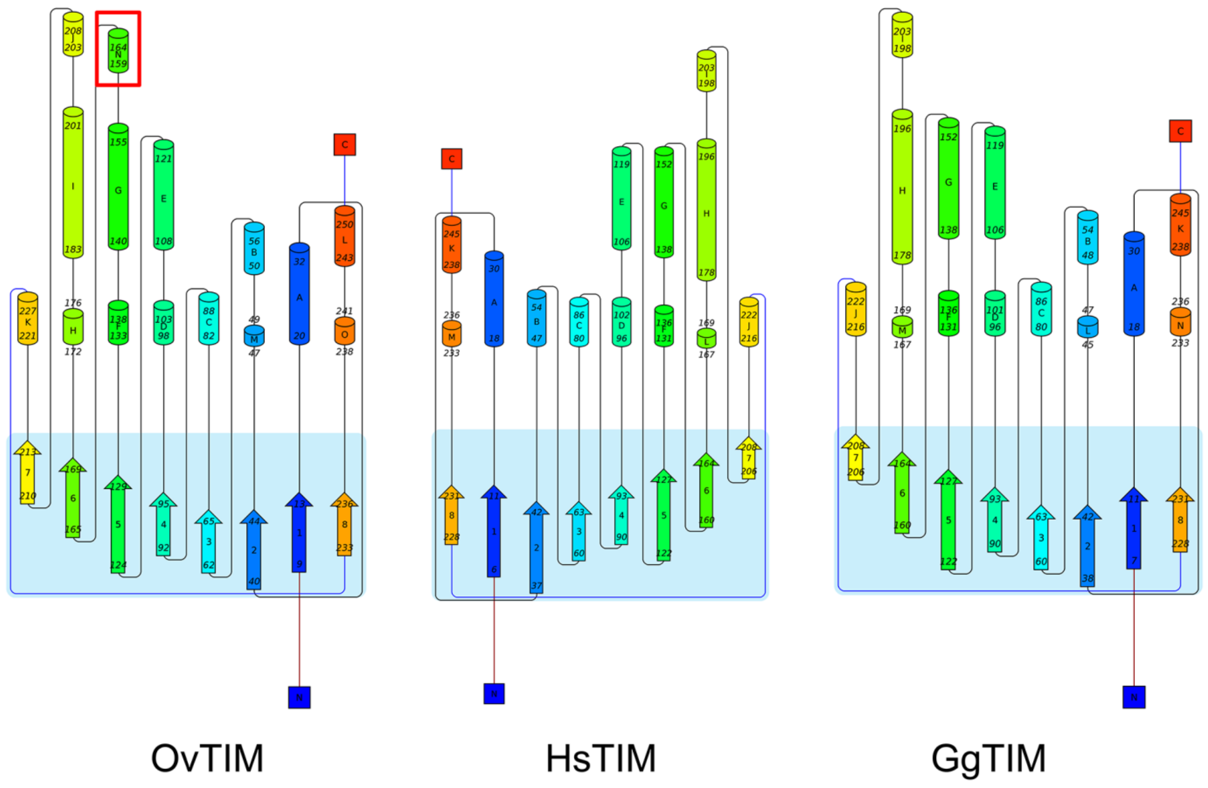

Supplementary Figure S2. Schematic topology of various TIMs, including OvTIM, HsTIM (PDB code: 4POC), and GgTIM (PDB code: 8TIM). The α-helices and β-sheets, which are labelled in the alphabetical order and the numerical order, respectively, are represented as cylinders and rectangular arrows. The residue numbers of the beginning and end of each secondary structure are written on the figure. The bundle of β-sheets is shaded in a *blue* rectangle, and the overall order of the secondary structures is shown in a rainbow spectrum from the N-terminal end (*blue*) to C-terminal end (*red*). The 3_{10}-helix N, which is represented as 3_{10}-helix γ2 in the manuscript, is designated as a *red* box.

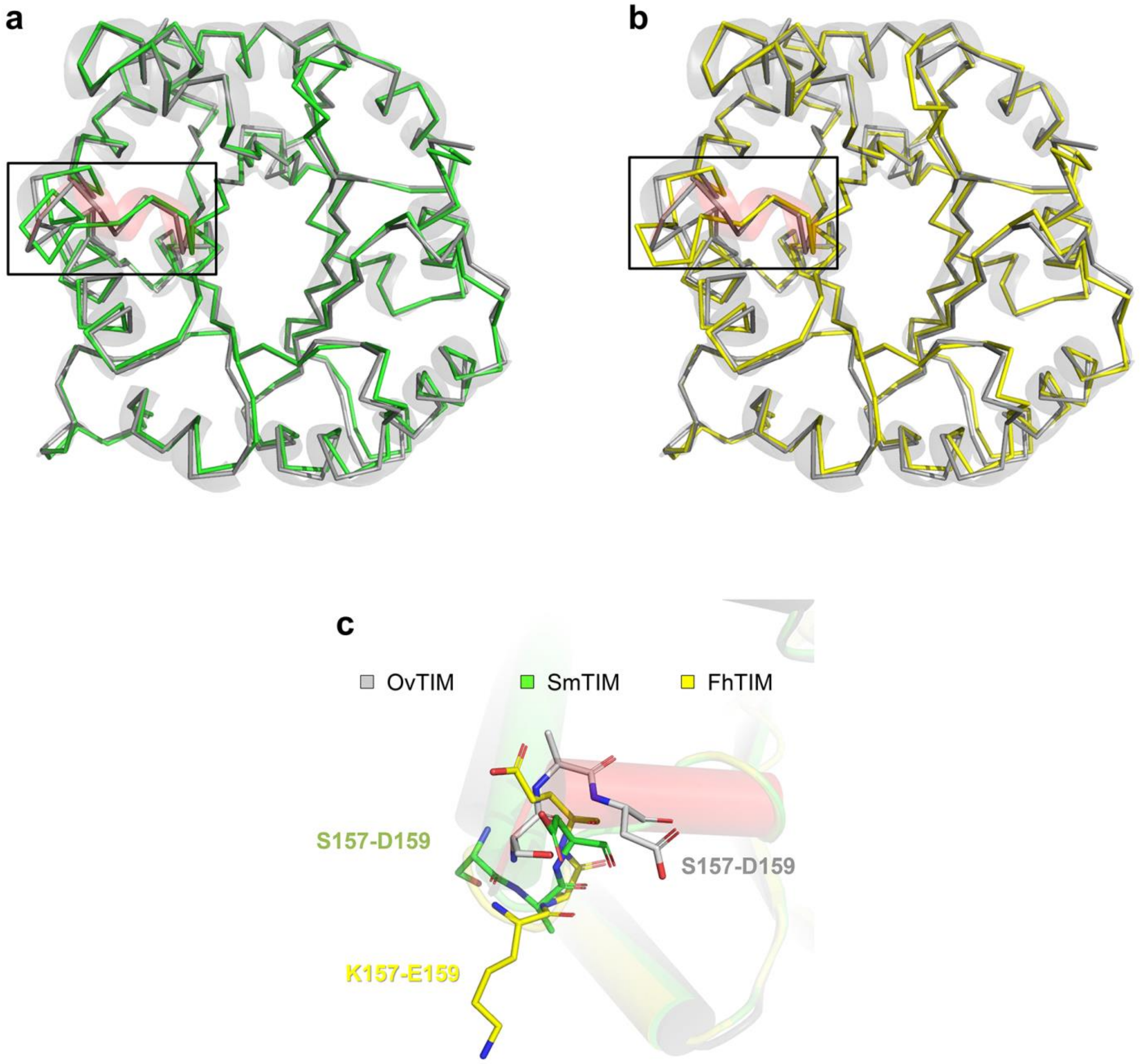

Supplementary Figure S3. (a, b) Superposition of OvTIM (*grey*) with *in silico* models of TIMs from *Schistosoma mansoni* (SmTIM, *green*) and *Fasciola hepatica* (FhTIM, *yellow*). Cα of three structures are represented as ribbon models and the cartoon model of OvTIM is additionally shaded as *black* colour for the visualization of the secondary structure. The SAD motif and 3_{10}-helix of OvTIM are designated as *black* box and highlighted as *red* colour. (c) Enlarged view of SXD/E motifs of three structures. The motifs are represented as stick models.

No	Name	Score	No	Name	Score	No	Name	Score	No	Name	Score	No	Name	Score
5	ARG	0.642	33	LYS	0.970	61	HIS	0.341	89	CYS	0.217	117	ALA	0.268
6	LYS	0.606	34	ILE	0.774	62	VAL	0.217	90	ASP	0.341	118	ILE	0.394
7	PHE	0.522	35	ASP	0.809	63	ALA	0.063	91	TRP	0.217	119	SER	0.555
8	PHE	0.321	36	PRO	0.882	64	ALA	0.028	92	VAL	0.051	120	ALA	0.441
9	VAL	0.341	37	ASN	0.809	65	GLN	0.014	93	ILE	0.033	121	GLY	0.441
10	GLY	0.195	38	THR	0.581	66	ASN	0.006	94	LEU	0.051	122	VAL	0.244
11	GLY	0.167	39	GLU	0.441	67	CYS	0.033	95	GLY	0.079	123	ASN	0.295
12	ASN	0.079	40	VAL	0.394	68	TYR	0.063	96	HIS	0.063	124	VAL	0.167
13	TRP	0.195	41	LEU	0.195	69	LYS	0.217	97	SER	0.142	125	ILE	0.120
14	LYS	0.110	42	VAL	0.217	70	VAL	0.244	98	GLU	0.079	126	ALA	0.167
15	MET	0.079	43	ALA	0.079	71	PRO	0.321	99	ARG	0.016	127	CYS	0.120
16	ASN	0.217	44	PRO	0.079	72	SER	0.295	100	ARG	0.167	128	ILE	0.295
17	GLY	0.268	45	PRO	0.051	73	GLY	0.167	101	HIS	0.268	129	GLY	0.368
18	SER	0.441	46	ALA	0.033	74	ALA	0.142	102	ILE	0.142	130	GLU	0.581
19	LYS	0.555	47	LEU	0.079	75	PHE	0.110	103	LEU	0.045	131	LYS	0.581
20	LYS	0.774	48	TYR	0.195	76	THR	0.016	104	LEU	0.167	132	LEU	0.843
21	GLU	0.581	49	LEU	0.120	77	GLY	0.000	105	GLU	0.063	133	GLU	0.949
22	ASN	0.394	50	PRO	0.268	78	GLU	0.008	106	THR	0.268	134	GLU	0.726
23	ASP	0.774	51	SER	0.441	79	VAL	0.016	107	ASP	0.368	135	ARG	0.843
24	LYS	0.809	52	VAL	0.341	80	SER	0.120	108	GLN	0.482	136	GLU	0.990
25	LEU	0.522	53	ARG	0.441	81	PRO	0.167	109	LEU	0.142	137	ALA	0.998
26	ILE	0.642	54	GLU	0.606	82	ALA	0.295	110	VAL	0.167	138	GLY	0.980
27	GLU	0.949	55	LYS	0.642	83	MET	0.134	111	GLY	0.321	139	LYS	0.949
28	MET	0.726	56	LEU	0.555	84	LEU	0.134	112	GLU	0.341	140	THR	0.726
29	LEU	0.606	57	ASP	0.774	85	LYS	0.394	113	LYS	0.110	141	GLU	0.882
30	THR	0.882	58	LYS	0.726	86	ASP	0.394	114	THR	0.244	142	GLU	0.911
31	HIS	0.980	59	ARG	0.642	87	VAL	0.295	115	ASN	0.482	143	VAL	0.606
32	ALA	0.911	60	PHE	0.522	88	GLY	0.368	116	HIS	0.321	144	CYS	0.522

No	Name	Score	No	Name	Score	No	Name	Score	No	Name	Score
145	PHE	0.726	179	THR	0.882	209	ARG	0.441	239	SER	0.321
146	ARG	0.606	180	ALA	0.703	210	ILE	0.441	240	LEU	0.441
147	GLN	0.394	181	THR	0.843	211	ILE	0.268	241	LYS	0.642
148	MET	0.482	182	GLU	0.809	212	TYR	0.394	242	PRO	0.809
149	GLU	0.726	183	GLN	0.911	213	GLY	0.268	243	ASP	0.726
150	ALA	0.502	184	GLN	0.809	214	GLY	0.368	244	PHE	0.482
151	ILE	0.394	185	ALA	0.606	215	SER	0.522	245	ILE	0.703
152	ARG	0.774	186	GLN	0.726	216	VAL	0.482	246	GLU	0.843
153	LYS	0.726	187	GLU	0.843	217	THR	0.673	247	ILE	0.522
154	ASN	0.555	188	VAL	0.606	218	ALA	0.809	248	CYS	0.555
155	LEU	0.606	189	HIS	0.522	219	ALA	0.882	249	ASN	0.774
156	SER	0.809	190	LEU	0.809	220	ASN	0.703	250	ALA	0.703
**157**	**SER**	**0.882**	191	ALA	0.774	221	CYS	0.673	251	ASN	0.726
**158**	**ALA**	**0.843**	192	VAL	0.581	222	ARG	0.843	252	ALA	0.911
**159**	**ASP**	**0.843**	193	ARG	0.673	223	THR	0.843			
160	MET	0.581	194	ARG	0.949	224	LEU	0.606			
161	TRP	0.555	195	TRP	0.774	225	ALA	0.642			
162	ASN	0.673	196	MET	0.703	226	LYS	0.882			
163	HIS	0.502	197	GLU	0.970	227	GLN	0.726			
164	ILE	0.341	198	GLU	0.990	228	PRO	0.774			
165	VAL	0.244	199	LYS	0.949	229	ASP	0.642			
166	ILE	0.321	200	VAL	0.911	230	VAL	0.522			
167	ALA	0.217	201	SER	0.949	231	ASP	0.502			
168	TYR	0.394	202	PRO	0.970	232	GLY	0.368			
169	GLU	0.217	203	ALA	0.937	233	PHE	0.368			
170	PRO	0.482	204	VAL	0.726	234	LEU	0.142			
171	VAL	0.441	205	ALA	0.726	235	VAL	0.268			
172	TRP	0.673	206	LYS	0.911	236	GLY	0.268			
173	ALA	0.606	207	SER	0.673	237	GLY	0.394			
178	LYS	0.937	208	ILE	0.522	238	ALA	0.482			

Supplementary Figure S4. The possible epitopes of the OvTIM dimer structure were predicted with the ElliPro web server using the following input parameters: Minimum score (0.6) and Maximum distance (6 Å). For clarity, the individual epitope prediction scores for one protomer in OvTIM are listed above. The scores of the SAD motif are highlighted in *red*.

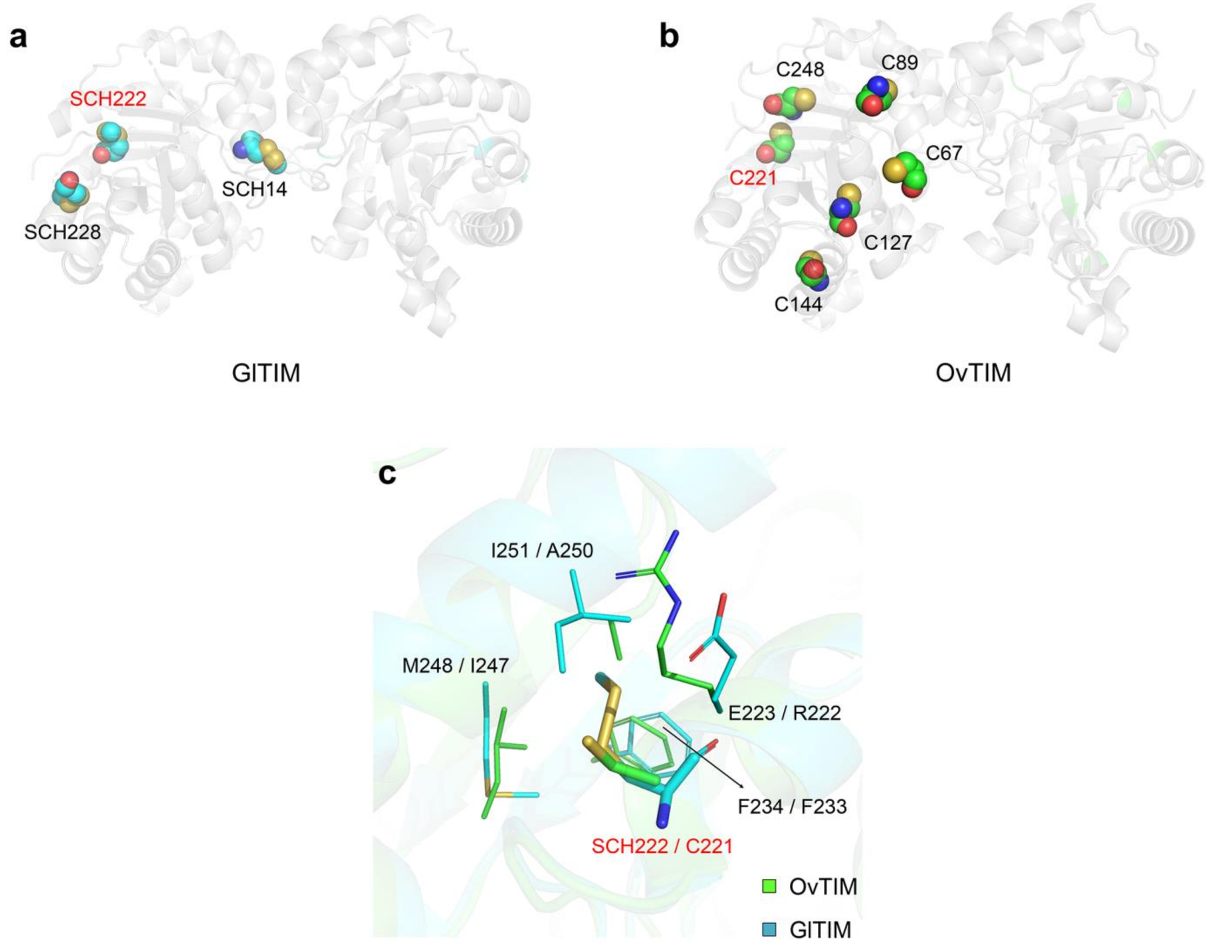

Supplementary Figure S5. (a) Derivatized Cys residues (SCH) of chemically modified *Giardia lamblia* TIM structure (PDB entry: 3PF3) are represented as *cyan* sphere models and SCH222 residue is labelled as *red* colour. (b) Cys residues that can be chemically modified in OvTIM are represented as *green* sphere models and C221 residue is labelled as *red* colour. (a, b) For clarity, the residues of only one protomer are shown and those of another protomer are highlighted in cartoon model. (c) Superposition of GlTIM with OvTIM. The SCH222 of GlTIM and C221 of OvTIM are represented as thick stick models and the surrounding residues that form the hydrophobic pocket are represented as relatively thin stick model. The labels of residues are written in GlTIM / OvTIM order.

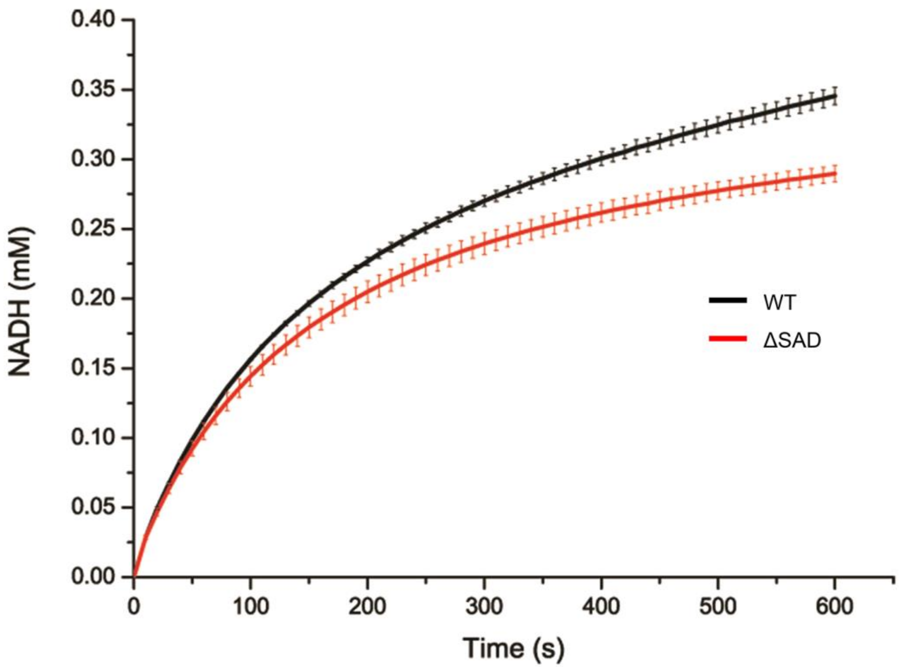

Supplementary Figure S6. Comparison of catalytic activity between wild-type and ΔSAD variant of OvTIM. Final reaction mixture contains 0.4 mM dihydroxyacetone phosphate (DHAP) and 0.018 mM OvTIM dimer. The measurement was conducted at 27 °C and 2 sets of activity curve are averaged and plotted.
